# Ten Simple Rules for Starting a Regional Student Group

**DOI:** 10.1371/journal.pcbi.1003340

**Published:** 2013-11-21

**Authors:** Avinash Kumar Shanmugam, Geoff Macintyre, Magali Michaut, Thomas Abeel

**Affiliations:** 1 Department of Computational Medicine and Bioinformatics, University of Michigan Medical School, Ann Arbor, Michigan, United States of America; 2 NICTA Victoria Research Laboratory, University of Melbourne, Melbourne, Victoria, Australia; 3 Computational Cancer Biology, Netherlands Cancer Institute, Amsterdam, The Netherlands; 4 VIB Department of Plant Systems Biology, Ghent University, Ghent, Belgium; 5 Broad Institute of MIT and Harvard, Cambridge, Massachusetts, United States of America; University of California San Diego, United States of America

Student organizations are a great way to network and take a break from the rigors of the classroom. They provide a range of benefits beyond regular coursework and can be critical to having a well-rounded education. Many students are active in organizations at an undergraduate level, but the increased demands of a master's or PhD typically result in reduced participation at a graduate level. However, a student organization can equally provide benefits for a graduate student, especially if it is centered on the student's area of study. In this article, we focus on Regional Student Groups (RSGs). An RSG is a group of like-minded students across a geographical region with a common field of research. The group provides a support network and collaboration opportunities via a collection of individuals who “speak the same language.” The RSG concept was created by the International Society for Computational Biology Student Council to address the needs of students in the field of computational biology in each region. Currently, the RSG program consists of over 20 regional student groups worldwide. In this article, we provide ten simple rules for how to start a regional student group in the hope that others will start up similar groups around the world.

## Background

The International Society for Computational Biology (ISCB) Student Council (SC) is an organization dedicated to nurturing and assisting the next generation of computational biologists. The SC offers networking opportunities and soft skill training to scientists in bioinformatics who are in the early stages of their careers. This is achieved through a number of activities, including the long-running symposium series organized in conjunction with the Annual International Conference on Intelligent Sytems for Molecular Biology (ISMB) and the European Conference on Computational Biology (ECCB). While successful in uniting students across the globe, the international symposiums did not provide opportunities that directly addressed the needs of computational biologists at a local level. Thus, a RSG program was established.

Since 2005, the RSG program of the ISCB Student Council has provided the opportunity for local student groups to become affiliated with the SC to share experiences and resources (http://www.iscbsc.org/content/regional-student-groups). RSGs typically represent the student community of one country, although we also have examples of supranational and subnational RSGs. Over the course of the last seven years, they have continued to grow and flourish. With over 2,000 students across 23 countries, the RSGs are providing valuable initiatives to support and promote students in bioinformatics and computational biology at the local level.

RSGs fill the gap between the global ISCB and SC organizations and the institutional associations that exist at many universities. Computational biology is a cross-disciplinary field, and young researchers in bioinformatics are often spread across the country in different groups with sometimes only a few bioinformaticians per institute. Having a country- or statewide association provides the critical mass to organize meetings, share ideas, and interact with peers. RSGs have a big advantage compared to a global society in that they can leverage local strengths and needs to offer a tailored program, yielding high impact.

The following ten rules are guidelines on how to get started with your own local group of trainees to form a successful local bioinformatics association. These rules are derived from the SC's experience with setting up and guiding RSGs in 23 different countries over several years. The rules are kept as generic as possible so they can be beneficial to other multidisciplinary areas as well.

## Rule 1: Form the Right Team

Finding the right people to start your group is crucial for its success and survival. As with all volunteer organizations, the workload needs to be shared among individuals, and initially, work will need to be put in without immediate reward. Everyone involved must *want* the organization to succeed and must be willing to invest time and effort. Ideally, this group of people should include one or more persons who can take initiative and set things rolling; good communicators who can get other people excited about the group and get them involved; a visionary who sees where the group should go; and finally, people who can take responsibility for dotting the i's and crossing the t's. The right set of motivated people will see the group through the first year, and the eventual rewards will be worth the effort.

It is a good idea to have a formal leadership committee that will take responsibility for activities. A collective of hard workers allows for a dynamic and nimble team. This group of people should be as diverse as possible in terms of experiences, ideas, career stages, etc. In the beginning, it may be easier to plan with a small group of friends to get the project started, but it is essential to broaden your horizons as soon as possible to increase the chances of success. In addition, it is helpful to have at least one faculty advisor or senior mentor who serves in an advisory role. They will help with getting access to resources and people, and having a senior advisor provides your fledgling group with increased credibility and legitimacy.

Besides the leadership committee, which would typically consist of three to four people, there should be a larger core committee. Core committee members should be located around the country or region (different cities, different universities) to make the organization truly representative of the region. Again, this will help with getting access to various resources and provide inside contacts for advertising later on.

Be careful to find the right people. In the end, putting in the time and effort to find enthusiastic and motivated people will be well worth it.

## Rule 2: Have a Plan

The basic goal of all regional student groups is to create a student network and work towards providing activities that benefit members. To achieve this, it is important to identify the needs of the community. For example, what do potential members want: better industry contacts, better information sharing, more meetings, or a support network of peers? The community of interest can be engaged on the sidelines of conferences or other talks and events in your field. If there aren't any such opportunities to meet with the community in your region, you can solicit community feedback by e-mailing flyers to universities to put up on noticeboards. Interacting with the community can be as simple as chatting with your labmates or picking up the phone and talking to colleagues. It is all about getting out from behind your computer and talking to other people. Creating opportunities for community interaction should be on top of your to-do list.

Once these needs have been identified, a plan should be put in place to attempt to fulfill those needs. The plan should be thorough and concise—don't write a novel, but ensure that sufficient details are given so that the plan can be put into action. Following are three aspects that should be covered in the plan.

1) The practical plan: What concrete things does the group plan to do in the next year? These need to be outlined with clear deadlines and measurable progress milestones. An easy way to do this is to identify the end goals to be completed in the year and then work backwards from these deadlines to fill in the milestones needed to get there. Make sure all goals are obtainable.

2) The aspirational plan: In general, you want to answer a few strategic questions: What does the organization want to achieve in five to ten years? How will it impact the community? How is the group working towards these goals? This should be more about setting a philosophical tone of what the RSG should achieve and act as a wish list. Some of the goals you want may not be possible right now, but do not restrain yourself. This is where you can dream about the future.

3) The succession plan: *The king is dead. Long live the king!* Finding a replacement leadership team is one of the biggest hurdles faced by regional student groups. Students may graduate or relocate, or may just become otherwise busy with their academic lives. To build a robust student organization, you need to plan to deal with some level of volunteer turnover. This is especially critical for the leadership roles. In the past, some RSGs have gone dormant after the team members were unable to meet their obligations to the organization and did not plan for their succession. The next generation of organizers has to be recruited while the current team is still active, so thinking about a succession plan early is a good idea. At every event, devise a plan to recruit new members and give them new responsibilities. Eventually, some of these members will become part of the next leadership committee. Creating a core committee (see Rule 1) is also very useful in this respect because it allows you to have a pool of people that are essentially leaders in training, lets people grow into their role, and helps maintain continuity.

## Rule 3: Organize Events

Events are a great way to engage the community, increase visibility, and get new members. Working towards an event can really help bring your leadership team together. A good rule of thumb is to use a big event to gain high visibility and follow up with a number of smaller events to ensure people keep coming back; i.e. consolidate your community. The large event may be an annual symposium or workshop. The smaller events could be as simple as a bi-monthly journal club that rotates through various universities in your area or a social event over a meal with a beer or a glass of wine. What is better than yeast to make people talk and like each other?

You have to be creative in the types of events you organize, and there is no rule set in stone for what will work for your local demographic. The type of events that will work are very dependent on your local needs, so get input from the community and plan accordingly. Soft skill training—such as the art of presentation or techniques for scientific writing—is generally well-received because it is lacking in almost all curricula around the world, so workshops for those skills tend to draw a large audience. Programming-related workshops and/or competitions are quite popular in many regions, but there has been equal success with breakfasts, essay writing, or quizzes. Remember that many students participate in an event to gain experience that will give them an edge when they apply for a job. Consider running events that help students in the quest for a job in academia or industry. Be creative, listen to your members, and you will deliver some great events.

## Rule 4: Think Frugal

For a student group, raising funds for activities is always a challenge. Establishing relationships with institutes, companies, or national granting agencies creates opportunities for acquiring funding or support. Getting cold hard cash can be difficult. However, it can be easier to obtain support in kind: for example, using an auditorium or classroom for free, or getting free products from a company to use as prizes. Some speakers might be willing to cover their own travel for speaking at a student event, or a university may cover the expenses of a speaker if all university employees can attend. Seek out and utilize opportunities to get the most “bang for your buck.” For instance, partner your annual event with a popular conference in your region or country. This will help you save on venue costs and will guarantee an audience.

## Rule 5: Think about Resources and Logistics

Once the core team has been formed, the next step is turning the organization into a recognizable brand. Just like with a sports team, the team needs a name, a logo, a web presence, and mailing lists. The list goes on. Just remember it is not all about brainstorming crazy ideas for a name or having fun designing a cool logo. It is about ensuring successful communication. Many practicalities have to be considered when setting up modes of communication, but you can be creative in finding the required resources. Where do you put the website? At the home institute of one of the committee members? At a free commercial service like Google Groups or with a paid service? Weigh the alternatives. Along with a website, it is recommended to set up an online identity at various social media sites including LinkedIn, Twitter, and Facebook. Make sure that the entire leadership has access to these to update them.

For day-to-day operations, most planning happens online, so there is limited need for physical logistics like meeting rooms. Most RSGs use Skype or other conference call technologies for meetings.

Resources for events, however, are a completely different beast. Events generally need their own name, logo, and web page. The hardest part is getting the physical resources sorted. You may need to take care of meeting or conference rooms, a submission system, poster boards, A/V equipment, etc. (see Rule 4). The location of your event is going to be a big factor in its success. The venue should be readily accessible by your target audience. If the majority of people attending your meeting arrive by car, make sure there is plenty of (free) parking available; if they will come by public transport, make sure your venue has good connections and that service runs before and after the scheduled times of the event. You do not want to kill an extremely successful event with a two-hour walk home from a remote place at night in the rain…

Involving people who are local to the event venue often makes your life easier. They are your boots on the ground to get everything moving smoothly. They can walk up to anyone in person at the venue and may immediately know the right person to get any issues sorted out.

Last but not least, we are all human: if your event lasts more than an hour, you need to think about basic needs like food, drinks, and restrooms. Contrary to popular belief, scientists need more than just caffeine to get through the day.

Organizing a conference requires significant investment in terms of time and planning. It may be easier to start with something smaller that does not involve so much up-front work to get the ball rolling. A practical training workshop, a journal club, a social gathering, a quiz…the possibilities are endless.

## Rule 6: Promote the Organization

When getting started, create a flyer about your organization that you can send to universities with a bioinformatics program and ask for it to be put up on their notice boards. If you do not have funds to send hard copies by post, you can just e-mail a PDF copy and ask for it to be printed out and put up. This is easiest if you have a core committee member at the target university who can print it and put it up (see Rule 1) or, even better, present it themselves to the new students during a course. It may be useful to keep an up-to-date list of people responsible for master's or graduate programs that you can contact every year to remind them that your group exists and ask them to promote it to the new students.

Approach the organizers of popular conferences or meetings in your region to get a time slot to give a (short) presentation about your RSG during the meeting: for example, during the opening remarks or before or after a coffee break. Even a slide about your organization in the cycle of slides that are shown in the breaks between talks may be useful. Approach local science magazines or the science sections of newspapers about whether they could run an article about your RSG. You can also put announcements in institutional newsletters or magazines. However, make sure you have a single point of contact where people can find information about the organization, about future events, where to sign up for regular news, etc. (see Rule 5).

The best way to promote the society is by word of mouth. Get out there and talk to fellow students at conferences, workshops, meetings, etc. Not all students will remember to follow up. Make sure you get their contact information and follow up after the meeting. Consider creating a newsletter or just a periodic e-mail alert to help members keep up to date with what is happening. It is also useful to update nonmembers to give them an incentive to join. Until your RSG attains a critical mass of people where it can become somewhat self-sustaining, you will need to work to push this information out to the community and actively recruit people to join.

Once people are coming to your events, you need incentives to keep them coming. There are two main incentives that are easy to have in any event, regardless of topic or theme: CV lines and networking. First, paper and/or poster awards, travel fellowships, etc. are things that are easy to provide, but give distinct benefit to the winner(s) in that they can include these on their CV to further their career. Second, maintaining a good social component to your events will make sure that people attend even when the event does not have a theme that is directly related to their research.

## Rule 7: Document Decisions, Methods, and Events

This sounds very serious…and it is! Because you want your group to grow and live a long time, you need some serious documentation. A student group should expect to see a fair amount of turnover. As mentioned earlier, students get *real* jobs, get busy, or just move on to other things. Therefore, it is very important to document discussions, decisions, and operating methods to help maintain continuity. The simplest way to maintain records is to conduct discussions by e-mail or any other electronic medium that has automatic archives. It is useful to use a mailing list with all of the leadership team for discussions rather than using personal e-mails. Not only does this make it easier to look up information later, but it also promotes transparency. For any offline discussions, take down quick minutes and share them on the mailing list. As your group matures, you can move to records that are more systematic.

After a larger event, it may be worthwhile to report on it. On the one hand, this recognizes the people who put a lot of time and effort in the event, and on the other hand, it also helps to promote your group (see Rule 6). Various journals offer a means to publish meeting reports, and local newspapers are often happy to run a story on local events. Having regular reports on your events will help with recruiting more members and will assist in gaining support to further expand (see Rule 8).

## Rule 8: Find Support and Benefactors

Once you have the basics in place, you can start looking towards the future. Aim to create relationships with other organizations, be they companies, universities, or other professional societies, because they can help provide funding and/or material support. Being associated with an established organization can help you apply for grants to support your work, too. For example, the SC has a funding program for RSGs where up to five projects are funded each year. Finding monetary support is going to be challenging, and you will need to be frugal (see Rule 4) with whatever funds you get together.

Make sure to cultivate good relations with local professors and administrators; they are your keys to accessing resources: using rooms at the University and convincing renowned scientists to speak at your event, for example. These personal connections will help you tremendously with acquiring logistical support for your group. Getting connections to companies while fundraising may even prove to be beneficial when looking for a job later on.

## Rule 9: Be Inclusive and Reward Contributions

One important step to make sure your RSG is healthy is to involve as many people as possible in actively contributing roles, not just as participants in your events. You should think how to motivate and incentivize people. Find ways to be inclusive and integrate people in the organization.

Many people will start participating out of interest and self-motivation, but competing priorities can get in the way. You must think of ways to keep people involved in the long run. Proper recognition for effort and involvement will make sure people want to volunteer to do a job again. Make sure you thank everyone by name in front of the event participants—bonus points if you can mention some of their actual contributions. Make sure that key contributors to your event have a title, e.g. “program chair,” for the person putting the agenda together and organizing the abstracts. This ensures their work can be added as a line on their CV and enables them to justify the time spent on doing this job.

Allow people to be involved no matter how much time they can contribute. Not everyone will have the time to be involved in the RSG at a leadership level. Try to find avenues where they can still contribute to the organization with only a minor commitment. Some examples of low-commitment activities may include reviewing abstracts for a symposium, answering questions on the mailing list, and forwarding useful information to the mailing list. Sometimes these tasks can be done more efficiently by a few dedicated people, but splitting it up and letting more people contribute can help create a feeling of belonging and ownership within the community. Even though involving many people is not the most efficient in the short term, it will likely improve the success of the RSG in the long term. Some of these minor contributors could get motivated to take on more significant positions later on.

## Rule 10: Less Talking and More Doing

You do not want to get lost in translation. Do not spend all your time making grand schemes to start the ultimate RSG or organize the most spectacular symposium ever. It is extremely important to get started. While you should not rush in headfirst, you also should not spend too much time planning every single aspect and contingency in minute detail. You may plan forever and get nothing done. This fine balance is easily missed, and it happens all too frequently that groups are trapped in endless discussions without ever moving beyond talk. Just think of something small you can do and get it done. It is better to do something that you can later improve than to envision something amazing that will just never happen. *Release fast, iterate often.*

## Conclusion

In addition to the considerable benefits that an RSG can provide to a student community, the authors can also attest to the fact that being involved in setting up an RSG will be an educational, transformative, and rewarding experience.

We hope that this short guide on starting up and running regional student groups will help inspire young scientists to band together and build the next generation of computational biologists.

